# Awake and Sleep Bruxism Among Israeli Adolescents

**DOI:** 10.3389/fneur.2019.00443

**Published:** 2019-04-26

**Authors:** Ephraim Winocur, Tal Messer, Ilana Eli, Alona Emodi-Perlman, Ron Kedem, Shoshana Reiter, Pessia Friedman-Rubin

**Affiliations:** ^1^Department of Oral Rehabilitation, The Maurice and Gabriela Goldschleger School of Dental Medicine, Tel Aviv University, Tel Aviv, Israel; ^2^Academy Branch of Israel Defense Forces Medical Corps, Haifa, Israel; ^3^Department of Oral Pathology and Oral Medicine, The Maurice and Gabriela Goldschleger School of Dental Medicine, Tel Aviv University, Tel Aviv, Israel

**Keywords:** awake bruxism, sleep bruxism, adolescents, anxiety, stress, oral habits, alcohol consumption, TMD symptoms

## Abstract

**Introduction:** Sleep and awake bruxism are potential risk factors for oral hard tissue damage, failure of dental restorations and/or temporomandibular disorders. Identifying the determinants of sleep and awake bruxism among adolescents will enable development of preventive interventions for those at risk.

**Objectives:** To determine emotional, behavioral and physiological associations of sleep and awake bruxism among Israeli adolescents.

**Methods:** Two thousand nine hundred ninety-three Israeli high school students, from five different high schools in Israel, were approached in the classroom and requested to complete online questionnaires on sleep and awake bruxism, emotional aspects, smoking, alcohol consumption, oral habits, facial pain, and masticatory disturbances. The final study sample concerning awake and sleep bruxism included 2,347 participants.

**Results:** 1,019 (43.4%) participants reported not experiencing any form of bruxism (neither sleep nor awake), 809 (34.5%) reported awake bruxism, 348 (14.8%) reported sleep bruxism and 171 (7.3%) reported both sleep and awake bruxism. Multivariate analyses (Generalized Linear Model with a binary logistic dependent variable) showed that one of the prominent variables affecting the occurrence of sleep bruxism was anxiety (mild, moderate and severe anxiety, Odds Ratios (OR) of 1.38, 2.08, and 2.35, respectively). Other variables associated with sleep bruxism were stress (each point in the stress scale increased the risk of SB by 3.2%), temporomandibular symptoms (OR = 2.17) and chewing difficulties (OR = 2.35). Neck pain showed a negative association (OR = 0.086). Multivariate analyses for awake bruxism showed an effect of moderate anxiety (OR = 1.6). Other variables associated with awake bruxism were stress (each point in stress scale increased the risk of AB by 3.3%), high and low levels of facial pain (OR = 2.94 and 1.53, respectively), creaks (OR = 1.85) and oral habits (OR = 1.36). Sleep bruxism was found to be a predictor for awake bruxism, and vice versa. In both cases ORs were 8.14.

**Conclusions:** Among adolescents, sleep and awake bruxism are associated with emotional aspects as well as with facial pain symptoms and/or masticatory system disturbances. Awareness is recommended to decrease potential risks to teeth, dental restorations, and the masticatory system.

## Introduction

Definition of bruxism has been under debate for some time. In 2013 (1), an international group of bruxism experts issued a consensus proposal based on the concept that bruxism is “a repetitive jaw activity” which can occur during sleep (sleep bruxism- SB) or during wakefulness (awake bruxism–AB). In 2018, it was argued that AB is a masticatory-muscle activity which occurs during wakefulness and is characterized by repetitive or sustained tooth contact and /or by bracing or thrusting of the mandible (2). Such behavior does not necessarily include other behaviors that people engage during the day, such as lip biting, pen biting etc. Those are rather referred to as oral habits. SB is a masticatory-muscle activity during sleep, characterized as rhythmic (phasic) or non-rhythmic (tonic). Neither of the bruxism forms is defined as a movement disorder or a sleep disorder in otherwise healthy individuals (2).

According to both definitions (1, 2), bruxism is characterized by clenching or grinding of the teeth and/or by bracing or thrusting of the mandible. Bracing could be interpreted as forcefully maintaining a certain mandibular position; and thrusting as forcefully moving the mandible in a forward or lateral direction. Both activities are performed without the necessary presence of tooth contact. This addition to “classical” bruxism activities (viz. clenching or grinding of the teeth) accords with the current view that bruxism is not caused by anatomical factors such as certain characteristics of the occlusion, and with the emerging consensus that bruxism involves more than tooth contact (1). Thus, bruxism should not be considered as a disorder, but as a behavior that can be a risk (and/or protective) factor for certain clinical consequences (2).

It was suggested that sleep and awake bruxism are positively associated, whereby, individuals reporting sleep bruxism have a higher probability of also reporting awake bruxism than individuals not reporting sleep bruxism (3, 4). However, these findings should be considered with care. Since sleep bruxism occurs during sleep, the report may reflect false negative proportion due to poor sensitivity of the assessment question(s).

Furthermore, a possible association between bruxism (sleep and/or awake) and temporomandibular signs and symptoms, especially pain, has been suggested. A generally accepted theory claims that masticatory muscle pain results from awake activity, rather than from sleep activity, while it is muscle stiffness when waking up in the morning, which may be associated with sleep bruxism (5).

Uncertainty exists concerning the causes, mechanisms and effects of bruxism. Even the reported prevalence of bruxing activities has a very large range (2.7–57.3% for awake bruxism, 4.1–59.2% for sleep bruxism) (6). According to Manfredini et al. (6), an accurate estimation of bruxism is problematic due to different diagnostic strategies, non-representative populations and comorbid conditions that may act as confounding variables. Additionally, dentally-based diagnosis of treatment and/or prevention demanding bruxism is not accurate in the absence of control for other potential causes of tooth wear (e.g., functional, endogenous, or exogenous factors).

The influence of stress and psychological factors in the etiology of bruxism is also controversial. Some (7) claim that awake bruxism is influenced by psychological factors, with no evidence to such relation with sleep bruxism. Others (8) are of the opinion that anxiety and stress are risk factors for sleep bruxism. Even the association of bruxism with demographic, behavioral and psychological risk factors is under dispute (7, 8).

It is, however, important to acknowledge that bruxism (sleep and awake) can pose a potential risk factor for negative oral health consequences such as painful temporomandibular disorders (TMD), mechanical tooth wear, prosthodontic complications, and others (3, 9).

The present study aimed to identify some of the factors that are associated with bruxism in general (not necessarily demanding treatment and/or prevention), among Israeli adolescents.

## Methods

The Chief Investigator of the Israeli Ministry of Education gave the ethical approval for the study and allowed its performance among students of five high Schools in Israel, located in different cities/areas.

Following coordination with the schools' administration, all students were met in the classroom by one of the researchers (T.M.). The researcher had no personal acquaintance with any of the students, nor access to their personal data.

Students received a full explanation about the study's aims and importance and were encouraged to participate. They were assured that the study was completely anonymous and that they were free not to participate without any consequences to their studies. Following the explanation, the school authorities sent a link to the students' mobile phones or personal computers, through which they could download the online questionnaire. At this point, students who chose to participate signed up an informed consent form and completed the questionnaires online using their mobile devices. To assure anonymity responses were automatically collected into a single database that did not enable tracing of the individual source.

### Questionnaire

The questions included in the final questionnaire were derived from the following sources:

- The official Hebrew version of the DC/TMD (10) (https://ubwp.buffalo.edu/rdc-tmdinternational/tmd-assessmentdiagnosis/dc-tmd/).
- Oral habits and temporomandibular noises were derived from a study by van Selms et al. performed among Dutch adolescents (9). The Dutch team tested the reliability of the questionnaire. The questionnaire was translated from Dutch to Hebrew and backwards and used in a previous study performed among Israeli population (4). The questions were multiple-choice and referred to the last month (9)
- The General Anxiety Disorder-7 (GAD-7) questionnaire (11)
- The Perceived Stress Scale-10 (PSS) questionnaire (12)

The studied variables were as follows:

1. **Facial pain symptoms and/or masticatory system disturbances** (during the past month **(TM symptoms**), were evaluated through the official Hebrew version of the DC/TMD (10) (https://ubwp.buffalo.edu/rdc-tmdinternational/tmd-assessmentdiagnosis/dc-tmd/). One was considered as suffering from TM symptoms when s/he gave a positive reply (a “yes” response) to at least one of the following questions:
  - Do you suffer from pain in your face, jaws, the front of an ear, or inside the ear**? (Facial pain)**.
  - Have you had pain in your neck? (**Neck Pain**)
  - Do you experience any difficulty in chewing? (**Chewing difficulties**)
2. **Bruxism** was assessed by the following questions (3, 9):
  - Sleep bruxism (**SB**)—“Have you been told, or did you notice by yourself, that you grind your teeth or clench your jaws when you are asleep?” (Yes, no, don't know)
  - Awake bruxism (**AB**)—“Have you been aware that you are clenching or grinding your teeth in wakefulness?” (Yes, no, don't know or unaware)
3. **Smoking** (3, 9) was assessed by the question: “Do you smoke cigarettes at present?” (Never, Occasionally, Regularly, Often, or Daily). For the purpose of this study, one was considered a smoker (a “yes” response) when one marked at least a regular smoking frequency (namely, marked one of the following responses: Regularly, Often, or Daily).
4. **Alcohol Consumption** (3, 9) was measured using the question “Do you drink alcohol at present?” (Never, Occasionally, Regularly, Often, or Daily). Alcohol consumption was considered positive (a ”yes“ response) when one marked at least a regular consumption (namely, marked one of the following responses: Regularly, Often, or Daily).
5. **Oral Habits** (3, 9) were evaluated by questions about various activities (Never, Occasionally, Regularly, Often, or Daily*)*. A habit was considered positive when the activity was marked as either–Regularly, Often, or Daily.
6. **Temporomandibular joint noises** (3, 9) **(Joint Noise)** while opening/closing the mouth, or while chewing was considered positive when a positive reply was given to at least one of the following questions:
  - Does your jaw make a clicking or popping sound when you open or close your mouth, or while chewing?'
  - Does your jaw make a scraping or grating sound when you open or close your mouth, or while chewing?'
7. **Anxiety-** the GAD-7 questionnaire (11), was used to classify and rate general anxiety disorder and assesses its severity in clinical practice and research. The questionnaire was initially developed, following DSM criteria, to screen generalized anxiety disorder and measure the severity of symptoms, *in the last 6 months*. GAD-7 is a 7-item measure that can be self-completed or administrated by an interviewer. Participants are asked how often over the past 2 weeks they have been bothered by each one of the seven cores items (e.g., worrying too much about different things; feeling afraid as if something awful might happen; not being able to stop or control worrying). Each item is assessed on a 1 to 4 Likert scale from (1 = not at all, to 4 = nearly every day). The cutoffs were adjusted to adolescents by Mossman et al. (13). Answers were scored by the index and divided into four severity levels: 0 = *None* (no anxiety whatsoever), 1 = *Mild anxiety*, 2 = *Moderate anxiety*, and 3 = *Severe anxiety*.
8. **Stress** – The PSS-10 questionnaire (12) was used to measure participants' perception of stress. The scale has been used with both adolescents and adults (14). Participants were asked to mention how regularly they experience stress, or a specific feeling, following different incidents in the last month. Each item is rated on a 5-point scale of 0–4 (“*Never*,” “*Almost Never*,” “*Sometimes*,” “*Fairly Often*” and “*Very Often*”). The total score ranges from 0 to 40. The scale was analyzed as a continuous variable.

### Statistical Analysis

Descriptive statistics followed by univariate Chi (2) or Fishers' Exact Test and *T*-Test analyses for PSS associated with AB and SB. The significance level was set to α = 0.05. For multiple comparisons of column proportions, the Bonferroni method for adjusted *p*-value was calculated. Regression results were also corrected using Bonferonni.

Significant results from the univariate analyses were used for further multivariate analyses using General Linear Model (GLM) with binary logistic dependent variables SB and AB. The reference group was non-bruxing participants (reporting neither AB nor SB). The reference group for GAD-7 score adapted for adolescent's independent variable was “No anxiety whatsoever.” Odds Ratio compared to the reference level in each categorical independent variable where the study groups SB or AB respectively, set as the risk category (of having either AB or SB). A Receiver Operating Characteristic (ROC) analysis was followed by Youden's J statistics to capture the maximum sensitivity and specificity performance of a dichotomous diagnostic test for PSS cut points predicting SB and AB.

The data were analyzed using IBM SPSS statistics version 23.0. (SPSS, Inc., Chicago, IL USA).

## Results

Overall 2,993 adolescents were approached, with a response rate of 88%. The initial study population included 2,634 students from five different high schools in Israel, as detailed in Table 1 (1,344 girls, 1,255 boys and 35 who did not specify their sex). The average age of participants was 15.7 years (with a Standard Deviation of 1.1 years).

**Table 1:** Final sample by age, sex, and school.

**School num**.	School location^*^	n^**^	Average age ± SD^***^	**Sex**		
				**Girls**	**Boys**	**Un-specified**
1	South	369	16.2 ± 1.0	195	169	5
2	Center1	1,046	15.4 ± 1.2	543	491	12
3	Center2	423	15.3 ± 1.0	191	224	8
4	East	451	15.6 ± 0.9	250	197	4
5	North	345	16.1 ± 0.9	165	174	6
Total		2,634	15.7 ± 1.1	1,344	1,255	35

* School location: South – next to Gaza strip; Center1- Ramat Gan; Center 2-Tel Aviv; East- Jerusalem; North- Kibutz Yagur.

** n, Number of students observed;

*** SD, Standard Deviation.

**Figure 1:**
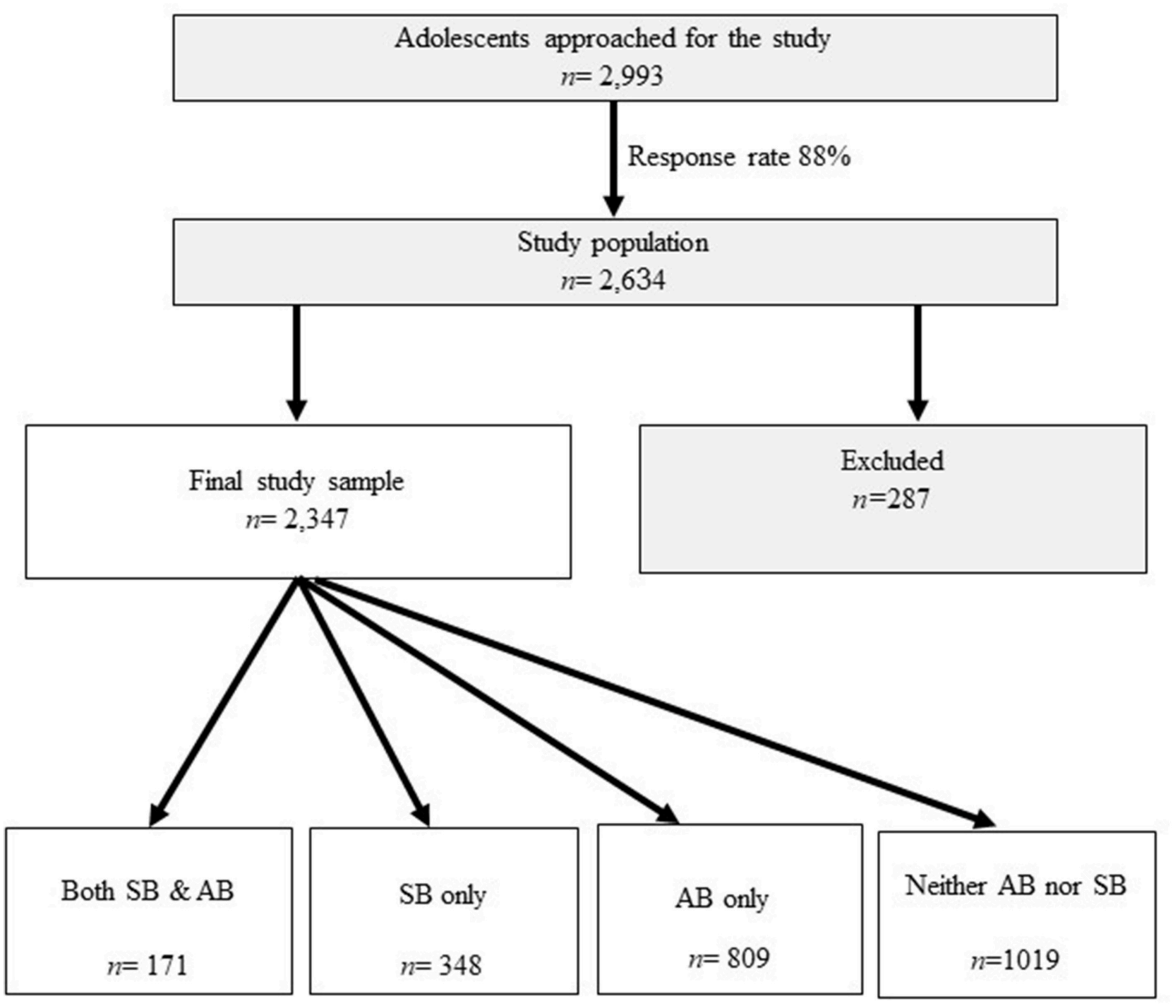
Flow chart of study groups.

Of the sample, 287 participants either did not respond to the questions regarding bruxism (*n* = 37) or indicated not knowing of any form of bruxism (neither sleep nor awake, *n* = 250), and were excluded from the analysis. The final study sample was 2,347, of whom 1,019 (43.4%) reported not experiencing any form of bruxism (neither sleep nor awake). This group was set as the reference group. 171 (7.3%) participants reported experiencing both AB and SB; 809 participants (34.5%) reported experiencing AB and 348 participants (14.8%) reported experiencing SB (Figure 1).

Since no significant differences were found between individuals who indicated not knowing of any form of bruxism, *n* = 250) and the final study sample (*n* = 2,347) regarding sex (*p* = 0.133), and age (*p* = 0.076), no adjustments for sex and age were introduced in the analyses of the final study sample.

Initially, participants who had undergone orthodontic treatment in the past, or were undergoing such treatment at the time of the study (45.9% of the final study sample), were analyzed as a separate group. As no significant differences were found among participants with and without orthodontic experience, the groups were aggregated and analyzed as one.

The most frequent symptoms associated with dysfunction of the masticatory system were neck pain (46.6%), followed by orofacial pain (28.2%), joint noises (21.2%). Pain and difficulties in chewing were less common (8.4%).

Table 2 presents frequencies of oral habits among the final study sample. Ninety percent of the participants reported chewing gum, with about 22% doing it very often. In addition, there was a high frequency of nail biting, pen chewing, and lip/cheek biting. The frequencies of alcohol consumption and smoking on at least a regularly base, were 8.5 and 5%, respectively.

**Table 2:** Frequencies of SB, AB, oral habits, smoking and alcohol consumption, by sex.

	***n***	**Total**	**Girls**	**Boys**
**SB**				
No	1,312	80.0%	75.9%	84.6%
Yes	327	20.3%	24.1%	15.4%
**AB**				
No	1,501	68.9%	67.9%	69.9%
Yes	678	31.1%	32.1%	30.1%
**Chewing gum**				
Never	284	10.8%	8.4%	13.2%
Sometimes	953	36.3%	27.5%	45.6%
Regularly	216	8.2%	10.4%	5.9%
Often	590	22.5%	24.0%	21.2%
Very often	579	22.1%	29.7%	14.0%
Total	2,622			
**Nails biting**				
Never	1144	43.6%	47.2%	40.0%
Sometimes	701	26.7%	29.8%	23.4%
Regularly	183	7%	4.9%	9.2%
Often	296	11.3%	8.5%	14.1%
Very often	297	11.3%	9.6%	13.3%
Total	2,621			
**Pen biting**				
Never	1602	61.2%	53.8%	68.9%
Sometimes	646	24.7%	29.9%	19.0%
Regularly	98	3.7%	3.7%	3.9%
Often	140	5.3%	6.8%	3.8%
Very often	132	5.0%	5.7%	4.4%
Total	2,618			
**Cheek/lip biting**				
Never	571	21.8%	14.7%	29.7%
Sometimes	1129	43.2%	42.1%	44.3%
Regularly	240	9.2 %	10.3%	7.9%
Often	405	15.5 %	19.6%	11.0%
Very often	269	10.3%	13.3%	7.1%
Total	2,614			
**Smoking**				
Never	2380	90.8%	92.8%	88.7%
Sometimes	141	5.4%	4.5%	6.4%
Regularly	28	1.1%	0.6%	1.5%
Often	31	1.2%	1.0%	1.4%
Very often	41	1.6%	1.1%	2.1%
Total	2,621			
**Alcohol consumption**				
Never	1,628	62.3%	65.1%	59.0%
Sometimes	784	30.3%	28.6%	31.5%
Regularly	81	3.1%	1.9%	4.5%
Often	105	4.0%	4.1%	3.9%
Very often	17	0.7%	0.3%	1.0%
Total	2,615			

At least 60% of the participants (final study sample) reported different degrees of anxiety and stress. Severe anxiety was found in 10.3% of the participants; moderate anxiety in 15.9% of the participants and mild anxiety among 32.7% of the participants.

A univariate analysis of associations between SB and the study variables is presented in Table 3. SB was associated significantly with the following variables: sex, joint noise, masticatory system symptoms, anxiety, oral habits, neck pain, difficulties in chewing, joint noises, and stress. Most of the variables, which showed a significant result for SB, showed also a significant result for AB (Table 4).

**Table 3:** Chi^2^ univariate analysis for SB by sex, age, and study variables.

**Independent variable**	**Category**	***n***	**Percent**	P^*^
Sex	Boys Girls	790 849	15.4^b^ 24.1^a^	<0.001
Age	14 15 16 17 18	263 405 487 401 83	19.4^a^ 19.3^a^ 21.8^a^ 19.2^a^ 15.7^a^	ns
Joint noises	Yes No	397 1,255	28.7^a^ 17.1^b^	<0.001
TM Symptoms (at least one)	Yes No	404 1,249	30.2^a^ 16.7^b^	<0.001
Smoking	Yes No	69 1,581	26.1^a^ 19.7^a^	ns
Alcohol	Yes No	135 1,509	22.2^a^ 19.8^a^	ns
Anxiety (GAD child)	None^*^ Mild Moderate Never	731 559 224 112	11.5^a^ 20.4^b^ 34.4^c^ 43.8^c^	<0.001
Oral habits	Yes No	587 1,064	26.6^a^ 16.4^b^	<0.001
Facial pain	Yes No	122 1,525	38.5^a^ 18.5^b^	<0.001
Neck pain	Yes No	193 1,436	31.1^a^ 18.5^b^	<0.001
Chewing difficulties	Yes No	1,524 113	36.3^a^ 18.8^b^	<0.001

anxiety whatsoever.

* Following Bonferonni correction.

*^a,b,c^,denote a different adjusted significant of a proportion level of SB for each Independent variable*.

**Table 4:** Chi^2^ univariate analysis for AB by sex, age and study variables.

**Independent variable**	**Category**	***n***	**Percent**	P^*^
Sex	Boys Girls	1,081 1,098	30.1^a^ 32.1^a^	ns
Age	14 15 16 17 18	377 557 671 493 91	29.2^a^ 33.8^a^ 35.0^a^ 27.6^a^ 22.0^a^	0.009
Joint noise	Yes No	513 1,691	44.2^a^ 27.5^b^	<0.001
TM symptoms (at least one)	Yes No	604 1,601	45.5^a^ 26.0^a^	<0.001
Smoking	Yes No	84 2,116	38.1^a^ 31.1^a^	ns
Alcohol	Yes No	167 2,028	32.9^a^ 31.3^a^	ns
Anxiety (GAD child)	None Mild Moderate Severe	913 786 326 152	21.4^a^ 32.6^b^ 47.9^c^ 51.3^c^	<0.001
Oral habits	Yes No	833 1,370	39.6^a^ 26.4^b^	<0.001
Facial pain	Yes No	199 1,999	50.8^a^ 29.4^b^	<0.001
Neck pain	Yes No	286 1,890	44.4^a^ 28.5^b^	<0.001
Chewing difficulties	Yes No	2,003 182	50.5^a^ 29.6^b^	<0.001

* Following Bonferonni correction.

ns, non significant.

*^a,b,c,^denote a different adjusted significant of percent level of SB for each Independent variable*.

**Figure 2:**
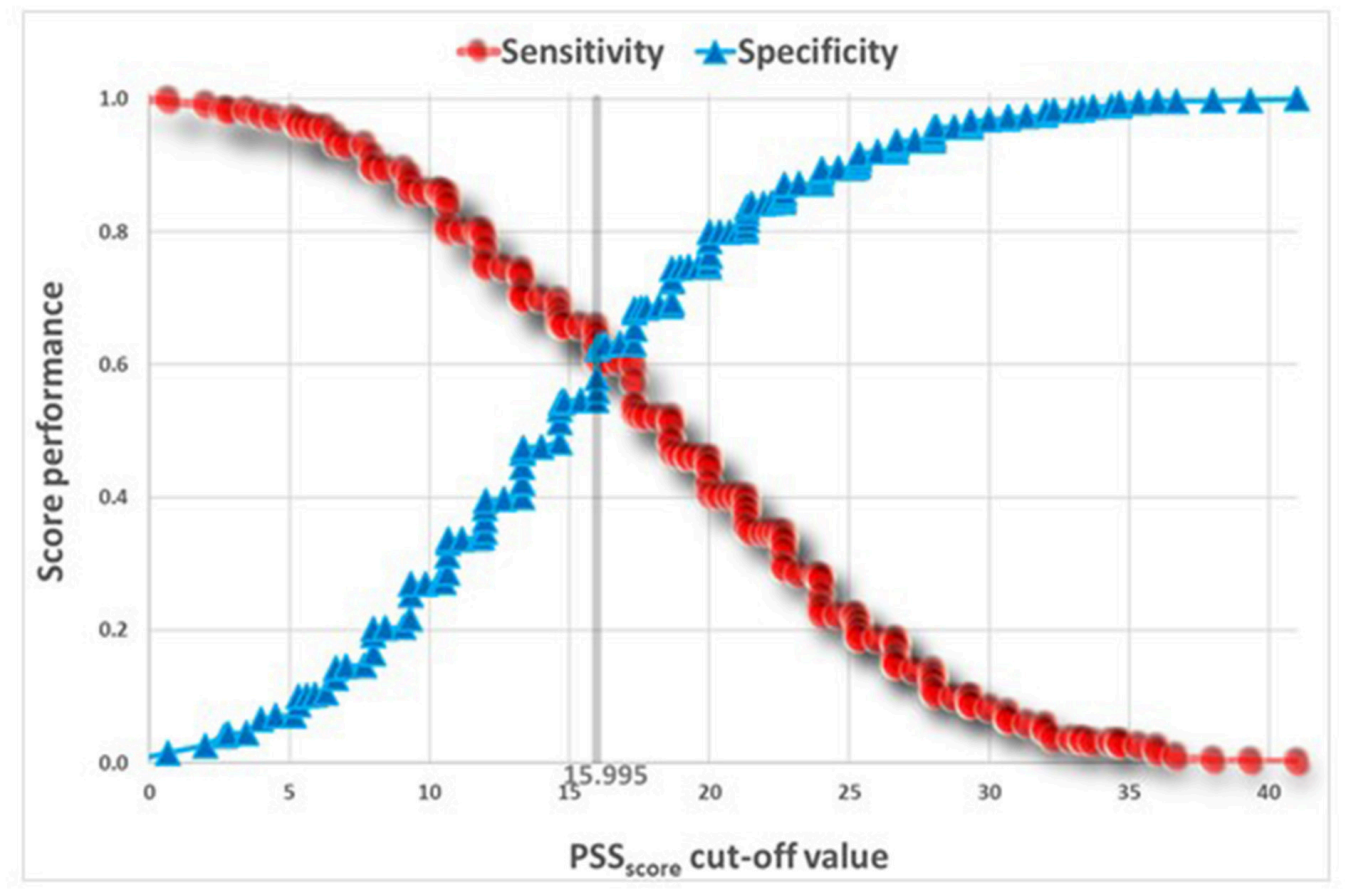
ROC Analysis of PSS predicting AB.

**Figure 3:**
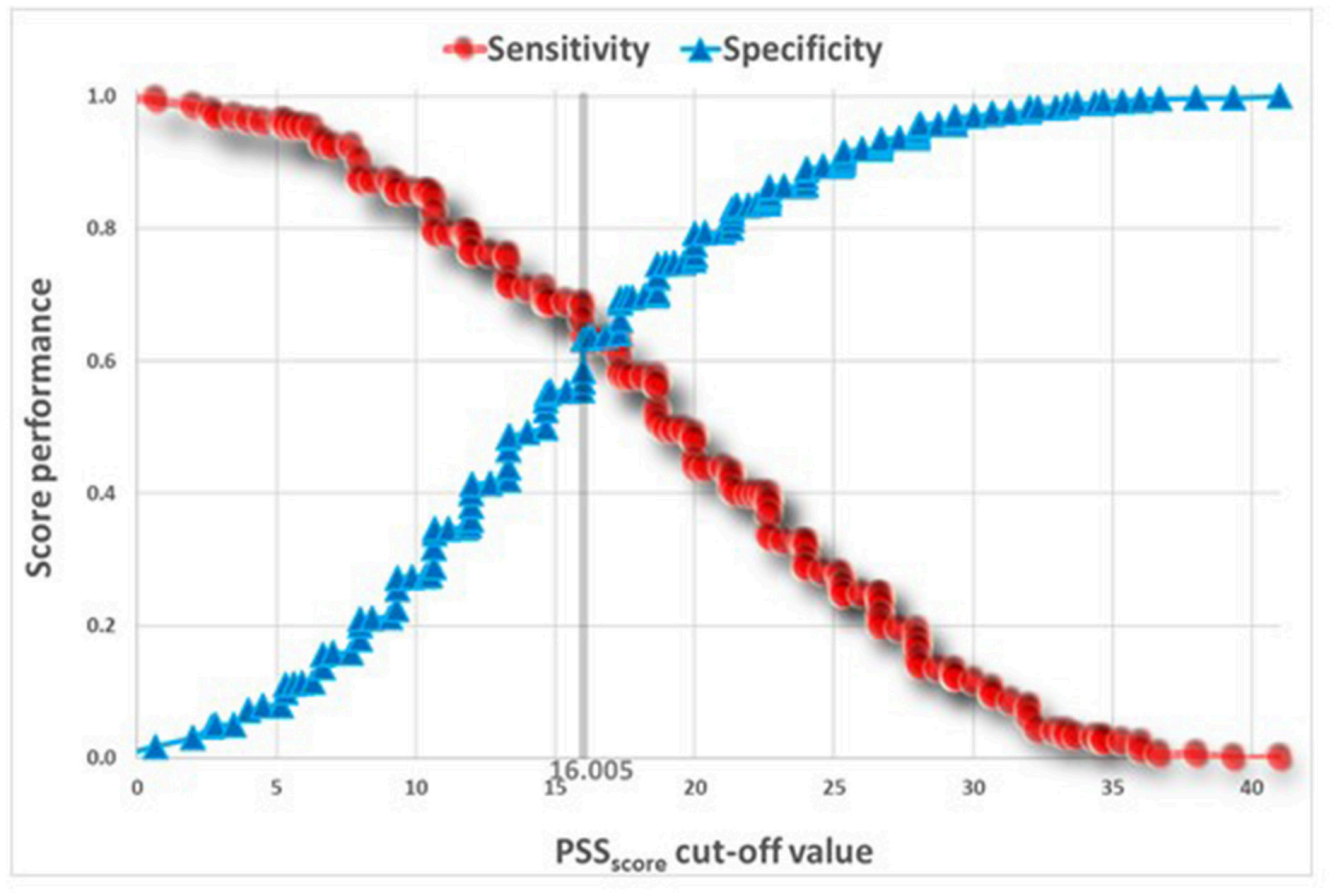
ROC Analysis of PSS predicting SB.

Table 5 presents *t*-tests univariate analysis of PSS for awake and sleep bruxism. The Reliability Statistics Cronbach's Alpha for PSS was 0.82 without need to delete any item. A Receiver Operating Characteristic (ROC) analysis captured a cut point of PSS ≥ 18.645 for SB, and a cut point of PSS ≥ 16.805 for AB (Figures 2, 3).

**Table 5:** *T*-Tests univariate analysis of PSS for AB and SB.

**Variable**	**Category**	***n***	**Average PSS ± SD**	***P***
SB	Yes	327	19.2 ± 8.4	<0.001
	No	1,310	14.8 ± 7.5	
AB	Yes	686	18.6 ± 7.8	<0.001
	No	1,500	14.9 ± 7.4	

### Generalized Linear Model (GLM) With a Binary Logistic Dependent Variable

Following univariate analyses, multivariate analyses were performed to evaluate which of the variables reaching significance affect the occurrence of SB and AB. Regression results also met significant criteria with Bonferonni adjustment.

Results of the multivariate analysis for SB are presented in Table 6. One of the prominent variables affecting the occurrence of sleep bruxism was anxiety (mild, moderate and severe anxiety; odds ratios of 1.38, 2.08 and 2.35, respectively, relative to None) with a positive linear trend according to anxiety level. Other variables correlated with SB were stress (each point of PSS scale increases the Odds Ratio of SB by 3.2%); TM symptoms (OR = 2.17) and chewing difficulties (OR = 2.35). Neck pain showed a negative correlation with SB (OR = 0.086).

**Table 6:** General linear model with sleep bruxism as a logistic binary dependent variable.

**Parameter**	***β***	**Hypothesis test**	**Exp(***β***)**	**95% Wald confidence interval for exp (*β*)**	
		**Sig**.		**Lower**	**Lower**
(Intercept)	−2.457	0.000	0.086	0.057	0.129
GAD-7 = Severe	0.855	0.006	2.352	1.278	4.331
GAD-7 = Moderate	0.731	0.002	2.077	1.304	3.308
GAD-7 = Mild	0.322	0.075	1.380	0.968	1.968
GADc-7 = None^a^	0a	1.000	1.000	–	–
PSS	0.031	0.008	1.032	1.008	1.056
Sex	0.131	0.472	1.140	0.798	1.675
TM symptoms	0.775	0.002	2.170	1.318	1.311
Joint noises	0.242	0.082	1.274	0.970	1.686
Oral habits	−0.014	0.959	0.986	0.577	4.331
Neck pain	−2.457	0.000	0.086	0.057	0.129
Chewing diff.	0.855	0.006	2.352	1.278	4.331

a *Reference value*.

The results of multivariate analyses for AB are presented in Table 7. Only “moderate” level of anxiety was found to affect the occurrence of AB relative to the reference “*None*” (OR = 1.6). Other variables increasing the occurrence of AB were stress (each point in PSS scale increased the occurrence of AB by 3.2%). Regarding Facial Pain, high level of pain (“*Very*”) increased the odds of AB by about 3 times and low level of pain (“*A little*”) by about 1.5 times (compared to “*None*”). No linear pattern was found since the level of ”*Moderate* pain” did not differ from the reference category. Creaks increased the odd of AB by 1.85 and oral habits by 1.36.

**Table 7:** General linear model with awake bruxism as a logistic binary Dependent variable.

**Parameter**	*****β*****	**Hypothesis test**	**Exp(***β***)**	**95% Wald confidence interval for exp (*********β*********)**	
		**Sig**.		**Lower**	**Lower**
(Intercept)	−1.108	0.124	0.330	0.080	1.356
GAD-7 = *Severe*	0.361	0.147	1.435	0.881	2.339
GAD-7 = *Moderate*	0.467	0.010	1.595	1.120	2.272
GAD-7 = *Mild*	0.182	0.162	1.200	0.930	1.549
GADc-7 = *None*^a^	0^a^	1.000	1.000	–	–
PSS	0.032	0.000	1.033	1.015	1.051
Age	−0.055	0.224	0.947	0.866	1.034
Facial pain = *very*	1.079	0.009	2.942	1.304	6.637
Facial pain = *Moderate*	0.342	0.114	1.408	0.921	2.153
Facial pain = *A little*	0.427	0.001	1.533	1.189	1.975
*Facial pain = None*	*0a*	*1.000*	*1.000*	–	–
Symptoms	0.174	0.324	1.190	0.842	1.683
Joint noises	0.214	0.550	1.239	0.614	2.499
Oral habits	0.304	0.004	1.355	1.105	1.661
Neck pain	0.103	0.195	1.108	0.949	1.295
Chewing diff.	0.031	0.876	1.032	0.695	1.532

a *Reference values*.

Sleep bruxism was found to be a predictor for awake bruxism, and vice versa. In both cases the ORs were 8.14 (95% IC = 6.12,10.83).

## Discussion

Sleep and awake bruxism are muscular activities with potential deleterious effects to the maxillofacial area (2). The present study aimed to identify some of the factors that are associated with bruxism among Israeli adolescents. This was an epidemiological study and the diagnosis of bruxism was based on self-report questionnaires, representing the lower (‘possible’) grade of bruxism diagnosis (1, 2). To achieve a definite diagnosis, use of polysomnography (for sleep bruxism) or electromyography (for awake bruxism) are needed. Regretfully, such tests are not feasible in an epidemiological study. Self –reported questionnaires are a common tool in large population studies like the present one (3, 6, 9, 15–17). Positive associations were found between questionnaire-based diagnoses of awake bruxism and diagnoses based on history taking combined with clinical examination (18). Furthermore, a meta-analysis showed medium to high specificity for questionnaires in the diagnosis of sleep bruxism (19).

In the present study, the prevalence of sleep bruxism was 14.8%, and that of awake bruxism 34.5%. While the results regarding sleep bruxism match prior data, these of awake bruxism are higher than previously reported in different societies (3, 6, 9, 20). The prevalence of sleep and awake bruxism were slightly higher than those reported by Manfredini et al. (6) for adults in a systematic literature review. A possible explanation for the differences may stem from the fact that adolescents in Israel reported relatively high rates of anxiety and a relatively high prevalence of oral habits (at least one type). Also, the differences among studies may origin from the assessment methods that might have led to differences in the reports of oral habits. Oral activities such as teeth clenching while awake, which are usually considered as part of AB, can sometimes be considered as oral habits and are not necessarily associated with clinical consequences.

Sleep bruxism was found to be a strong predictor for awake bruxism, and vice versa (OR 8.4). This is in accordance with a previous study (21) which showed that awake bruxism increases the odds of sleep bruxism 5-fold (and vice versa), suggesting that both entities have much in common. Manfredini and Lobbezoo (7), claimed that awake and sleep bruxism seem to be of different pathogenesis but are difficult to distinguish clinically. Possibly, participants perceive awake and sleep bruxism as a single entity a fact which unable satisfactory diagnosis through self-reported questionnaires. A large-scale investigation is warranted in an attempt to substantiate the complex relationship between sleep and awake bruxism.

Neither smoking nor alcohol consumption were associated with sleep or awake bruxism. The relation between alcohol consumption and sleep bruxism is controversial. While some studies (9) did not find an association, others (19, 22) reported associations between heavy smoking and heavy alcohol consumption and sleep bruxism. It is noteworthy that the distributions of alcohol and tobacco use in the present study were skewed. The population in the present study was young, and the number of smokers and alcohol consumers was small (only 3% reported smoking and 5% consumed alcohol often or very often). Alcohol consumption in Israel is the lowest in the OECD countries (https://www.timesofisrael.com/alcohol-consumption-in-israel-among-lowest-in-oecd-countries), so that the low percentage of alcohol consumers among the study population is not unusual. The young age of participants, together with the low percentage of smokers and alcohol consumers, may have led to the weak associations between these variables and sleep bruxism. The insufficient exposure of participants to smoking and alcohol unable reaching a meaningful conclusion.

The most prominent variables associated with sleep bruxism were anxiety and stress. The etiology of sleep bruxism seems to be centrally mediated (23). It occurs mostly during a switch from deep sleep to shallow sleep (24). It is reasonable that stress influences the quality and deepness of sleep, causing more switches between deep sleep to shallow sleep and secondarily aggravating the sleep bruxism. Additional factors increasing the risk of sleep bruxism were the presence of joint noises, facial pain symptoms, and the performance of oral habits. These symptoms are well-documented as being associated with bruxism (both sleep and awake) (25). Joint noises may be due to TMJ disc displacement with reduction caused by bruxism, based on a proposed etiology that frictional “sticking” of the disc is the cause of the disorder. In addition, the intra-capsular pressure performed during clenching may affect the joint lubrication and temporary anchorage of the disc. The energy needed to break adhesion of the disc is converted into the joint sound. For the performance of oral habits, the study suggests once again that they may be detrimental to the masticatory system (26). Another interesting finding was that sleep and awake bruxers reported significantly greater difficulties in chewing. This finding may be due to the fact that bruxers (sleep and awake) also reported significantly more facial and neck pain. In other words the chewing difficulties are probably secondary to the pain and not directly related to the bruxing activity.

In the multivariate model, awake bruxism was associated with moderate/ high anxiety and stress. This is in accordance with the common notion of the role of psychosocial factors, especially stress, in awake bruxism (6). Awake bruxism, is often claimed to be a response to stress and anxiety (27). As expected, AB shows higher sensitivity to stress than SB.

The ROC curves (Figures 2, 3) show that the discriminatory power of the PSS questionnaire is around 65% in both AB and SB, which is not a high performance. The PSS was originally intended to measure stress levels in adults. Nevertheless, it has been successfully used in previous studies to evaluate stress among adolescents (14, 28), a fact that led to its use also in the present study. Possibly, the questionnaire's adult norms are less appropriate for adolescents. Use of other measures, specifically developed for adolescents to assess stress, may have led to stronger results.

Additionally, the results show significant differences in the occurrence of SB between sexes (24.1% among girls vs. 15.4% among boys; *P* < 0.0001, Fisher's Exact Test). No such differences were found for AB (32.1 vs. 30.1%, accordingly). Sex differences in the occurrence of SB and AB are contradictory. Some studies report sex differences in the occurrence of bruxism among adolescents while others do not (3, 25, 29, 30). A systematic literature review (31) including 22 publications and accounting for more than 19,000 subjects aged 2 to 12 years, found that the prevalence of sleep bruxism in children was highly variable between the studies (3.5–40.6%), with a commonly described decrease with age and no gender differences. Thus, the findings reported above regarding sex differences in the occurrence of SB (or lack of it regarding AB) should be considered with care and need further examination.

Bruxism, both sleep and awake, can carry negative oral health consequences (e.g., severe masticatory muscle pain or temporomandibular joint pain) (1). Pain, if present, may be associated with changes in stress and anxiety. Accordingly, a vicious cycle develops. This cycle, if not interrupted promptly, may cause a neuroplasticity converting the pain into centrally mediated. Identifying factors that affect sleep and awake among adolescents will enable to better define treatment and/or prevention demanding bruxism and propose preventive interventions for subjects at risk.

Taking the findings together it can be concluded that bruxism (sleep and awake) among adolescents is associated with both emotional aspects, as well as with facial pain symptoms and/or masticatory system disturbances. Awareness to these aspects among adolescents can benefit our understanding of the bruxing behavior in order to prevent potential future negative effects.

## Ethics Statement

The Chief Investigator of the Israeli Ministry of Education gave the ethical approval to the study and allowed its performance among students of five high Schools in Israel, located in different cities/areas.

## Author’s Note

This study was undertaken in partial fulfillment of a DMD thesis at the School of Dental Medicine, Tel Aviv University, Tel Aviv, Israel.

## Author Contributions

EW: creation of the research concept, academic supervision of the DMD thesis, editing of the manuscript. TM: data acquisition and writing the DMD thesis. IE: academic supervision, manuscript preparation and revision, final approval of manuscript. AE-P: manuscript preparation and revision. RK: data analysis. SR: critical revision of the manuscript. PF-R: contribution to study concept design, analysis, and interpretation, drafting and critically revision of the manuscript, supervision of the DMD thesis. All authors read and approved the final manuscript.

### Conflict of Interest Statement

The authors declare that the research was conducted in the absence of any commercial or financial relationships that could be construed as a potential conflict of interest.
